# Diamond-Coated Silicon ATR Elements for Process Analytics

**DOI:** 10.3390/s21196442

**Published:** 2021-09-27

**Authors:** Nicolai Arndt, Carsten Bolwien, Gerd Sulz, Frank Kühnemann, Armin Lambrecht

**Affiliations:** Fraunhofer IPM, Georges-Köhler-Allee 301, D-79110 Freiburg, Germany; arndt.nicolai@web.de (N.A.); carsten.bolwien@ipm.fraunhofer.de (C.B.); gerd.sulz@ipm.fraunhofer.de (G.S.); frank.kuehnemann@ipm.fraunhofer.de (F.K.)

**Keywords:** infrared spectroscopy, ATR (attenuated total reflection), PAT (process analytical technology), inline, online, sapphire, silicon, diamond, nanocrystalline, acetonitrile, propylene carbonate

## Abstract

Infrared attenuated total reflection (ATR) spectroscopy is a common laboratory technique for the analysis of highly absorbing liquids or solid samples. However, ATR spectroscopy is rarely found in industrial processes, where inline measurement, continuous operation, and minimal maintenance are important issues. Most materials for mid-infrared (MIR) spectroscopy and specifically for ATR elements do not have either high enough infrared transmission or sufficient mechanical and chemical stability to be exposed to process fluids, abrasive components, and aggressive cleaning agents. Sapphire is the usual choice for infrared wavelengths below 5 µm, and beyond that, only diamond is an established material. The use of diamond coatings on other ATR materials such as silicon will increase the stability of the sensor and will enable the use of larger ATR elements with increased sensitivity at lower cost for wavelengths above 5 µm. Theoretical and experimental investigations of the dependence of ATR absorbances on the incidence angle and thickness of nanocrystalline diamond (NCD) coatings on silicon were performed. By optimizing the coating thickness, a substantial amplification of the ATR absorbance can be achieved compared to an uncoated silicon element. Using a compact FTIR instrument, ATR spectra of water, acetonitrile, and propylene carbonate were measured with planar ATR elements made of coated and uncoated silicon. Compared to sapphire, the long wavelength extreme of the spectral range is extended to approximately 8 μm. With effectively nine ATR reflections, the sensitivity is expected to exceed the performance of typical diamond tip probes.

## 1. Introduction

Infrared attenuated total reflection (ATR) spectroscopy is a common laboratory technique for the analysis of highly absorbing liquids or solid samples. When radiation in an optical material is totally reflected at an interface with a material of a lower index of refraction, part of the wave penetrates into the sample. The penetration depth of this evanescent wave, i.e., the probed sample thickness, is approximately equal to the radiation wavelength *λ_s_* in the sample [1,2]. In the mid-infrared (MIR) range (3 < *λ* < 20 µm), this is only a few µm. The ATR technique is used in many applications, e.g., in medical diagnostics [3,4], food quality analysis [5], beverage industry [6,7], water contamination monitoring [8,9], determination of moisture in transformer and lubrication oil [10], pharmaceutical process analytics [11], and monitoring of rubber polymerization [12].

However, ATR spectroscopy is rarely found in industrial processes where inline measurements, continuous operation, and low maintenance costs are key issues. Most materials for MIR spectroscopy and especially for ATR do not have either high enough infrared transmission or sufficient mechanical and chemical stability to be exposed to process fluids, abrasive components, and aggressive cleaning agents. Up to a wavelength of 5 µm sapphire is an established material [6,7,13]. For longer wavelengths diamond is most frequently used. However, because of the high costs only very small diamond elements, e.g., tiny crystals at the tip of fiber probes, are generally used [14,15]. These diamond crystals allow only a few ATR reflections, so that the sensitivity of such arrangements is reduced compared to planar sensors with larger ATR elements and correspondingly higher number of reflections. However, rugged fiber probes with a planar diamond tip allowing multiple ATR reflections are also available and have been successfully used for process analytics in a laboratory environment [16,17]. A compact multireflection ATR setup using a diamond disk with a diameter of 14 mm and a thickness of 1 mm was recently used for the online detection of hydrogen peroxide [18].

In the laboratory, ATR crystals made of other materials like silicon, germanium, and zinc selenide are used for the longer wavelength MIR range. However, these materials are not sufficiently robust for use in industrial processes, either chemically or mechanically [19]. This is especially the case for process analytical applications in chemical and food industries. Process instruments must cope with challenging cleaning-in-place (CIP)-processes where materials in contact with process media must withstand mechanical scrubbing as well as hot water vapor and cleaning acids and bases.

Thus, for compact and cost-effective ATR process sensors a less expensive ATR material compared to bulk diamond is needed. Next to the selection of a suitable material for the ATR element, the geometry of the sensor element and packaging are also important issues to prevent fouling and enable easy cleaning of the surface [13].

In the present work, we investigated how nanocrystalline diamond (NCD) coatings on ATR elements of silicon can be realized and how sensitive and robust those process sensors for MIR-ATR measurements can become. NCD coatings can be applied to silicon substrates using an effective manufacturing process. In addition to an increased chemical and mechanical resistance of the ATR element surface, the optical properties of the diamond top layer influence the optical behavior of the entire ATR element. This effect is investigated theoretically and experimentally. Subsequently, uncoated and coated Si ATR elements are used in a process spectrometer, ATR spectra of different fluids are recorded, and the results are evaluated. Finally, the use of diamond-coated ATR elements in compact process sensors is discussed and an outlook is given.

## 2. Materials and Methods

### 2.1. Preparation and Characterization of Nanocristalline Diamond Films on Silicon

Silicon hemispheres and ATR crystals (see Table 1), obtained from Alkor Technologies, St. Petersburg, Russia, were coated with nanocrystalline diamond (NCD) films using a Hot Filament CVD process at GFD GmbH, Ulm, Germany. Typical HFCVD process parameters using CH_4_ and H_2_ as main reaction gases were: substrate temperatures 750–900 °C, filament temperatures: 1900–2200 °C, chamber pressure 1–20 mbar, and growth rates 50–400 nm/h. Optimizing the process parameters, homogeneous NCD films with a thickness up to several µm were obtained. Further details on the HFCVD process can be found in the literature, e.g., [20,21].

**Figure 1:**
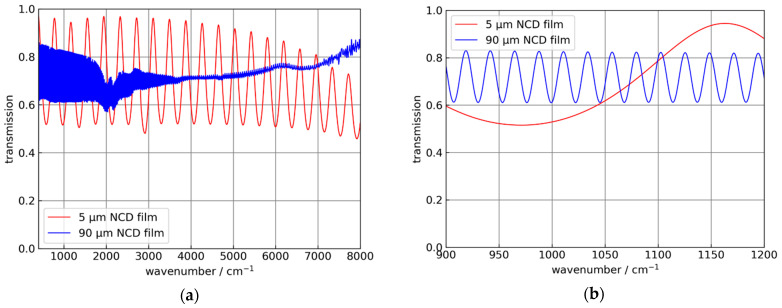
Transmission measurements of “thick” NCD films, which were used to determine the effective refractive index of the material. (**a**) Infrared transmission of 5 and 90 µm thick NCD films. (**b**) Detail showing the interference fringes of the 90 µm thick NCD film with higher resolution.

In the following, for the Si hemispheres and the Si ATR crystals a refractive index of 3.43 for Si in the MIR spectral range was used [22]. For the NCD films, a value of 2.38, which is typical for CVD-diamond materials, was used [23,24]. This value was confirmed by infrared transmission measurements of freestanding NCD films with 5 and 90 µm thickness, respectively, measured with a Bruker Vertex 80V FTIR spectrometer (Bruker Optik GmbH, Ettlingen, Germany), and shown in Figure 1.

For process applications, the coating on a Si ATR surface must not have any defects. For that reason, exemplary tests were performed by GFD: Visually defect-free NCD-coated Si samples were exposed to concentrated KOH, which is an established etching solution for Si. After rinsing and drying, the NCD coating was removed by an O_2_ plasma etching process. The formerly coated Si surface was then inspected for the occurrence of any etch pits by optical and scanning electron beam microscopy. In the case of pinholes in the NCD coating, such etch pits at the interface would have been formed by KOH intrusion. However, for NCD films with a thickness >300 nm no etch pits were observed, indicating that the original NCD coating was pinhole-free.

Additionally, the suitability of NCD-coated Si elements for applications in the beverage industry was investigated by CENTEC GmbH, Maintal, Germany. Uncoated and NCD-coated Si elements were exposed to 5% *v*/*v* NaOH solutions at 80 °C, 3% *v*/*v* HNO_3_ solution at 40 °C, and several beverages.

Subsequent visual inspection showed that the coated sides of the samples were not affected by the agents. Uncoated Si surfaces were not stable.

### 2.2. Theoretical Background and Simulation

#### 2.2.1. Attenuated Total Reflection (ATR)

**Figure 2:**
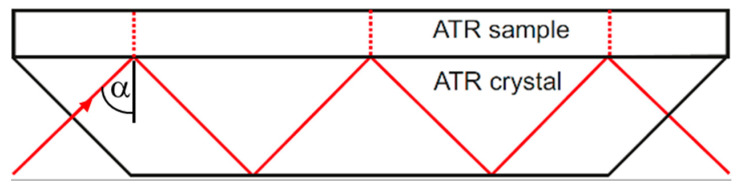
Typical ATR multireflection configuration.

The basic principles of the effect of attenuated total reflection (ATR) are explained in many textbooks, e.g., Harrick [2]. A typical configuration for multireflection ATR is shown in Figure 2. The angle of incidence *α* is higher than the critical angle *α_c_*, so that the wave is totally reflected at the interface.

In the ATR crystal, a standing wave is generated and because of the boundary conditions, the electromagnetic field decays exponentially into the sample. This field is called the evanescent field.

The critical angle *α_c_* can be defined as follows [25], where *n*_1_ and *n*_2_ are the real parts of the refractive indices of ATR crystal and sample, respectively.

(1) $$\alpha_{c}=\arcsin\left(\frac{n_{2}}{n_{1}}\right).$$

Because of the electromagnetic continuity conditions, the wave intrudes into the sample and decays exponentially. The penetration depth *d_p_* is defined as the distance from the interface when the amplitude of the evanescent wave is decreased to 1/e of the amplitude at the interface [25].

(2) $$d_{p}=\frac{\lambda }{2\pi \times \sqrt{{\left(n_{1}\right)}^{2}\times \sin{\left(\alpha \right)}^{2}-{n_{2}}^{2}}}.$$

Typically, the penetration depth of the evanescent wave is in the range of the wavelength *λ* of the radiation used. The wavelength-dependent absorption of the evanescent wave inside the sample leads to an attenuation of the reflected beam. By measuring the spectral dependence of the reflected beam intensity, an ATR spectrum is obtained.

For a standard transmission measurement, one obtains for the transmission factor *T* according to Beer’s law:

(3) $$T=\frac{I}{I_{0}}=e^{-a\times d}\;\mathrm{with}\;a=4\pi \times k/\lambda .$$

where *I*_0_ is the initial intensity, *I* denotes the intensity after passing the sample with the thickness *d*, *k* is the optical extinction coefficient, and *a* describes the absorption coefficient of the sample. With *k*, the complex refractive index *n* of a material can be expressed as:

(4) $$n=n^{\prime }+i\times k.$$

This also allows us to define the important term of absorbance:

(5) $$A=-\log_{10}\left(\frac{I}{I_{0}}\right)=4\pi \times k\times \frac{d}{\lambda_{0}\times \ln\left(10\right)}.$$

The absorbance *A* is a dimensionless quantity. However, in spectroscopy the AUs (absorbance units) and milli-absorbance units (mAUs) are commonly used. Throughout this paper we use this convention.

Compared to transmission spectroscopy, ATR spectroscopy has some special characteristics: the strength of the absorbance also depends on parameters such as incidence angle and the refractive indices of ATR material and sample.

Analogous to transmission spectroscopy, the effective penetration depth *d_e_* can be defined for ATR spectroscopy [25]. It describes the path length through the sample that is necessary to produce the same absorbance in a transmission experiment:

(6) $$d_{e}=\ln\left(R\right)\times \frac{\lambda_{0}}{4\pi \times k}.$$

where *R* describes the polarization-dependent reflection factor. For uncoated ATR crystals it is calculated in a straightforward way using the Fresnel equations [25]. By analogy to Equation (5), the absorbance for ATR measurements is obtained:

(7) $$A=-\log_{10}\left(R\right).$$

The extension for coated ATR crystals is shown in Section 2.2.2. The use of a multireflection configuration as in Figure 2 linearly increases the absorbance by the number of reflections.

**Figure 3:**
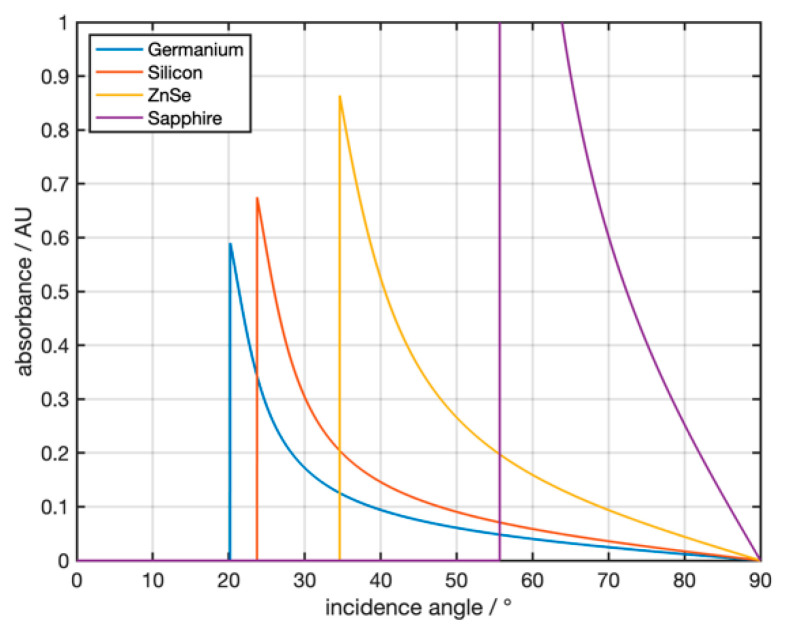
Theoretical absorbance of water at 3300 cm^−1^ for the ATR materials germanium, zinc selenide, sapphire, and silicon.

Figure 3 shows the theoretically calculated, angle-dependent absorbances for different ATR crystal materials and a water sample for a single reflection of infrared radiation at 3300 cm^−1^ (corresponding to the strong absorption band of water). The complex refractive index of the sample is 1.404 + 0.253i [26]. The simulation shows that both the crystal material and the angle of incidence have a strong influence on the absorbance.

#### 2.2.2. Simulations for ATR Elements with a Coating Layer

**Figure 4:**
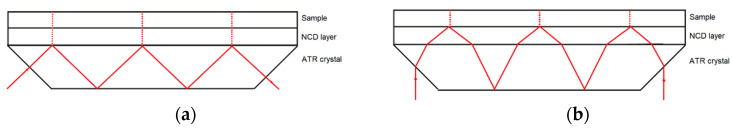
(**a**) Scenario 1: total internal reflection at the interface between ATR crystal and coating layer. A 45° wedged element with perpendicular incidence is shown. (**b**) Scenario 2: total internal reflection at the interface between coating layer and sample. To achieve a smaller incidence angle at the Si–NCD interface a 45° incidence angle at the air–Si interface is shown. The dimensions do not have the same scale.

The ATR effect is strongly changed by a covering layer on the ATR element. The optical properties of the coating material, the coating thickness, and the angle of incidence influence the measured absorbance of a sample. In the following, these influences for NCD layers on silicon are described by theoretical calculations. Depending on the incidence angle α, two different scenarios are possible (see Figure 4).

In scenario 1, the incident radiation is totally reflected at the interface between ATR crystal and diamond coating. Thus, the electromagnetic field in the diamond coating and in the sample is evanescent. According to Equation (1) for NCD layers on Si, this is the case for incident angles at the Si–NCD interface α ≥ 44°. In scenario 2, the incident wave intrudes into the diamond coating and is totally reflected at the interface between diamond coating and sample. In this case, the evanescent field is only present in the sample. According to Snell’s law and Equation (1) for NCD layers on Si and a water sample this is the case for incident angles at the Si–NCD interface 23° < α < 44°.

Subsequently, it is interesting to investigate the influence of an NCD coating on the absorbance of the ATR measurement. The coating leads to an increase or decrease of the absorbance caused by interferences in the coating. The absorbance for scenario 1 can be determined by using numeric simulation software, e.g., the Lumerical 3D/2D Maxwell’s Solver for Nanophotonic Devices [27].

**Figure 5:**
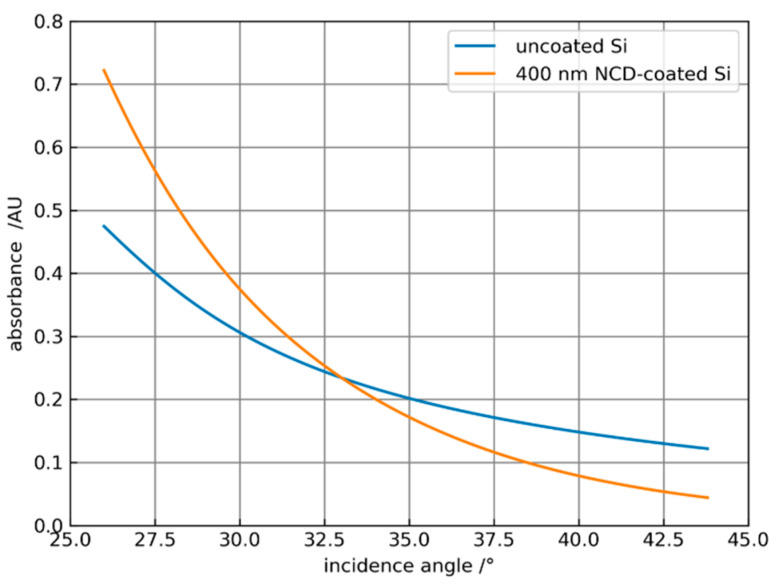
Dependence of the water absorbance at 3300 cm^−1^ on the angle of incidence for uncoated Si and NCD-coated Si with a thickness of 400 nm.

In the following, only scenario 2 is considered, in which the absorbance is calculated analytically using Equation (7). However, the reflection factor R for a coated ATR system is different from the reflection factor of an uncoated ATR system. The corresponding equations can be found in [28]. In Figure 5, the calculated absorbances of an uncoated Si ATR crystal and a Si ATR crystal with a 500 nm thick NCD coating are shown for the strong water absorption band at a wavenumber of 3300 cm^−1^. For the refractive indices of silicon, diamond, and the sample, the values 3.43 [22], 2.38 [23,24], and 1.404 + 0.253i [26] are used, respectively.

**Figure 6:**
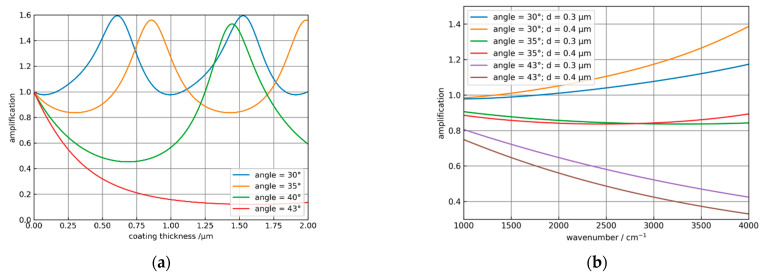
(**a**) Dependence of the amplification on the coating thickness for water at 3300 cm^−1^ and incidence angles α of 30°, 35°, 40°, and 43°. (**b**) Spectral dependence of the amplification for α = 30°, 35°, and 43° and two different NCD coating thicknesses for propylene carbonate (PC).

Obviously, the coating leads to an amplification of the absorbance for angles smaller than 33°. As the angles increase, the decrease in the absorbance of the coated system is stronger than for the uncoated element. In Figure 6a the amplification = absorbance_coated_/absorbance_uncoated_ is plotted against the coating thickness of the ATR crystal for different incidence angles for water with the same parameters, as in Figure 5. Obviously, it is possible to optimize the coating thickness for a specific angle of incidence or vice versa to maximize the amplification. Figure 6b shows the spectral dependence of the amplification for the solvent propylene carbonate (PC) with an index of refraction of 1.41 for different angles of incidence and 300 and 400 nm thick NCD coatings. Here, amplification is only possible at an angle of 30° and increases toward higher wavenumbers. For α > 35° an increasing attenuation is calculated. Corresponding measurements of ATR spectra of PC are reported in Section 3.2.

### 2.3. Experimental ATR Setup Using Si Hemispheres

**Figure 7:**
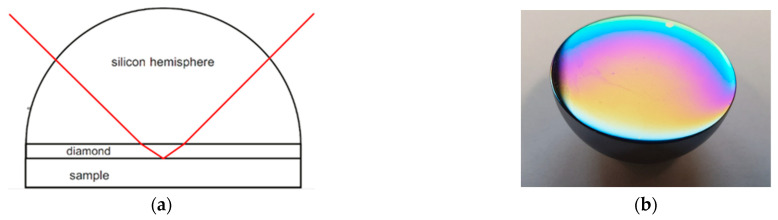
(**a**) Optical path in a hemispherically shaped and NCD-coated ATR element. The diamond layer is shown enlarged for visibility. (**b**) Image of an NCD-coated Si hemisphere.

To perform variable-angle ATR spectroscopy, a “Seagull” ATR accessory setup (Harrick Scientific Inc., New York, NY, USA) was attached to a Bruker Vertex 80V FTIR spectrometer (Bruker Optik GmbH, Ettlingen, Germany). With this setup it was possible to set the angle of incidence in a range between 5° and 85°. The ATR elements were hemispheres in order to use this range of possible angles. In Figure 7a the optical path in such a hemispherical ATR crystal is shown. Figure 7b shows the planar surface of a coated silicon hemisphere. The thin-film interference indicated that the coating thickness was not uniform. Since the optical beam was mainly reflected at the center of the basal plane predominantly the coating thickness at the center was relevant for the measurements. The coating thickness can be determined from the interference pattern of, e.g., a normal incidence transmission or reflection spectrum. With respect to film inhomogeneity a 10% uncertainty of the thickness determination was estimated.

### 2.4. Experimental ATR Setup Using Planar Si ATR Elements

With the “Seagull” setup, only one ATR reflection can be realized. Planar ATR elements with multiple reflections (as sketched in Figure 2) can be used to provide a larger absorbance signal for a given sample. This is important for process applications, where a more compact and robust design is required, and measurements are generally performed using a fixed incidence angle.

**Figure 8:**
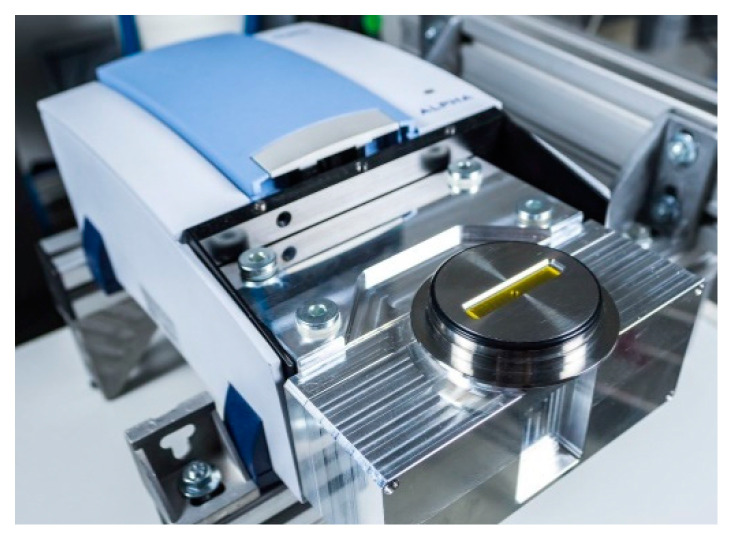
Planar ZnSe-ATR element with Varivent flange coupled to a Bruker Alpha FTIR instrument.

Therefore, planar Si ATR elements with a 45° wedge for radiation in- and outcoupling (see Table 1) were mounted into a Varivent flange, which is frequently used for instrumentation in the beverage industry. Figure 8 shows how the flange was coupled to an Alpha FTIR spectrometer (Bruker Optik GmbH, Ettlingen, Germany). With such ATR elements, 11 ATR reflections can be achieved nominally. Furthermore, other ATR materials such as sapphire or ZnSe can be easily mounted into the Varivent flange for comparison [13]. Results of the spectroscopy measurements with the planar Si ATR elements C0, C1, C2 are reported in Section 3.2.

The entire unit (flange and spectrometer) can be coupled to a process line for continuous operation. Alternatively, the Varivent flange assembly may be coupled to a compact photometer or micro-spectrometer module to realize a compact process sensor [13]. Such a sensor with a sapphire ATR element is available, for example, from CENTEC GmbH, Maintal, Germany, to measure the CO_2_ content in beverages [7].

## 3. Results

### 3.1. Results from Uncoated and NCD-Coated Hemispheres

**Figure 9:**
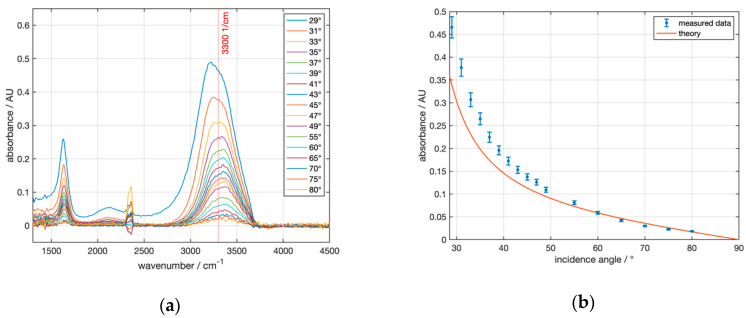
ATR absorbance spectra of water recorded with the Seagull setup using the Si hemisphere H0 with varying angles of incidence. (**a**) shows the water absorbance spectra with reference to air at the respective angle, smoothed by a moving average filter and offset-corrected to 4000 cm^−1^. (**b**) compares the angle-dependent absorbance values at 3300 cm^−1^ to theoretical values.

With the “Seagull” setup, angle-dependent ATR measurements with uncoated and coated silicon ATR crystals H0, H1, H2 were performed. Figure 9a shows the absorbance of water measured with the uncoated hemisphere H0 at angles of incidence of 29° and above. The distinct water absorption band at 3300 cm^−1^ is clearly visible.

Qualitatively, there was a fair agreement of the experimental data with the model calculation in Figure 3 for uncoated Si. Figure 9b shows that almost all measured absorbances were above the theoretically predicted values. These deviations became very large, especially at small angles, i.e., close to the critical angle. One possible reason is that close to the critical angle, owing to the divergence of the infrared beam, a considerable portion of the radiation was lost by transmission, resulting in a reduced intensity of the reflected beam and, hence, an apparently increased absorbance.

**Figure 10:**
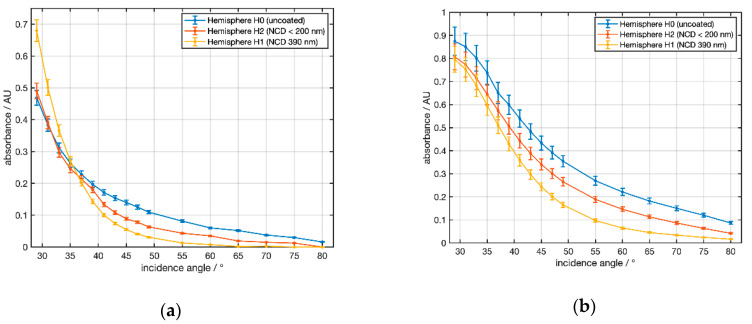
Experimentally determined angular dependence of the absorbance for silicon as ATR material and two NCD coatings with two samples: (**a**) water at a wavenumber of 3300 cm^−1^ and (**b**) propylene carbonate at a wavenumber of 1782 cm^−1^. Data points are connected by lines to guide the eye.

The experimental results of the angle-dependent ATR measurements of the water absorption band at 3300 cm^−1^ with NCD-coated silicon hemispheres are shown in Figure 10a for water as a sample. For angles of 29° < α < 34° an increase in the absorbance compared to H0 was observed for hemisphere H1. For hemisphere H2 no amplification effect was observed. Above an angle of 34°, however, the absorbance of both diamond-coated silicon hemispheres H1 and H2 was lower than for H0. In Figure 10b the measurement was repeated with the solvent propylene carbonate (PC) at a strong absorption band at 1782 cm^−1^. Here, diamond coating led to an attenuation of the absorption for all angles. The attenuation increased at larger angles.

To compare the experimental observations shown in Figure 10 with the theoretical results (Figure 5 and Figure 6) selected data are shown in Table 2. For water at 3300 cm^−1^ the experimental value of the amplification exceeded the result of the simulation at all angles. For PC @ 1781 cm^−1^ the simulation predicted a small amplification at 30° that was not observed in the experiment. However, for 35° and 43° the simulation fit well to the measured data.

Regarding the inhomogeneity and the estimated 10% uncertainty of the thickness of the NCD films the experimental data gave a qualitative confirmation of the calculated angular dependence of the amplification in both settings.

### 3.2. Results with Planar ATR Elements

With the setup described in Section 2.4, measurements with planar uncoated (C0) and coated (C1, C2) Si elements were performed. Sample liquids were dripped onto the surface of the ATR elements. To prevent evaporation of the liquid the wet surfaces were covered with microscope lids during the measurements.

#### 3.2.1. ATR Spectra of Water

**Figure 11:**
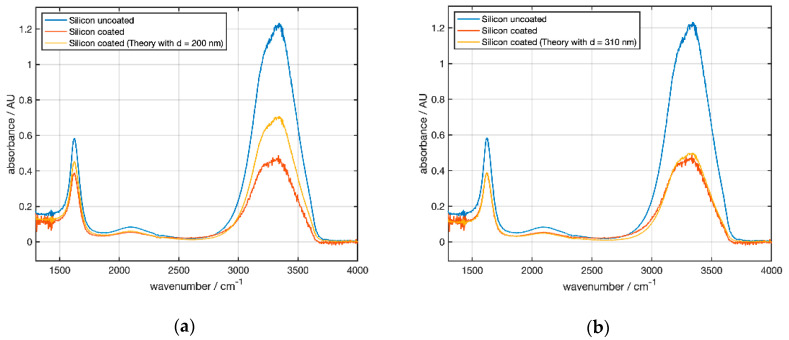
ATR spectra at 45° for water measured with the alpha spectrometer. Blue curve: measurement of uncoated Si Element C0. Red curve: measurement of coated element C1. The orange curve shows a simulated spectrum obtained by multiplication of the blue curve by a wavelength-dependent attenuation factor. (**a**) A coating thickness of 200 nm is used for the simulation. (**b**) Using a coating thickness of 310 nm an optimum fit of simulated and measured spectra is obtained.

Figure 11a gives an absorbance of 1.23 at 3300 cm^−1^ compared to 0.14 for the uncoated hemisphere C0 at an angle of 45° (Figure 10a), which is a factor of 8.8 because of multiple reflections.

According to Figure 10 one would expect an attenuation of the absorbance for a 200 nm thick NCD coating to ≈ 0.4 at 3300 cm^−1^ (see Table 3). Figure 11a shows that for C1 both peaks (3300 and 1600 cm^−1^) showed a smaller absorbance than the simulation for a layer thickness of 200 nm. This indicated that the real layer thickness was larger than reported by the supplier (see Table 1). Extrapolating the simulated spectral dependence of the attenuation factor (Figure 6b) to 45° allowed us to adjust the coating thickness. The best agreement was obtained for 310 nm (Figure 11b).

The increased noise below 1500 cm^−1^ was caused by low transmission of the Si substrates because of intrinsic absorption. At 3300 cm^−1^ noise resulted from low transmission owing to the strong liquid water absorption. Additionally, atmospheric water vapor in the setup had sharp absorption lines between 3300 and 4000 cm^−1^, which also contributed to the measured spectra.

#### 3.2.2. ATR Spectra of Acetonitrile and Propylene Carbonate

For further investigations, solutions of acetonitrile (AC) in propylene carbonate (PC) were used. To study the feasibility of an ATR setup, spectroscopy of AC solutions is an alternative to pressurized dissolved CO_2_, which is important for the beverage industry and to toxic isocyanates, which are key chemicals with high-volume production worldwide [13]. Acetonitrile has a strong absorption at 2252 cm^−1^.

Additionally, propylene carbonate has a strong absorption band at 1782 cm^−1^, which is out of reach of the frequently used sapphire ATR elements. AC and PC are miscible liquids.

**Figure 12:**
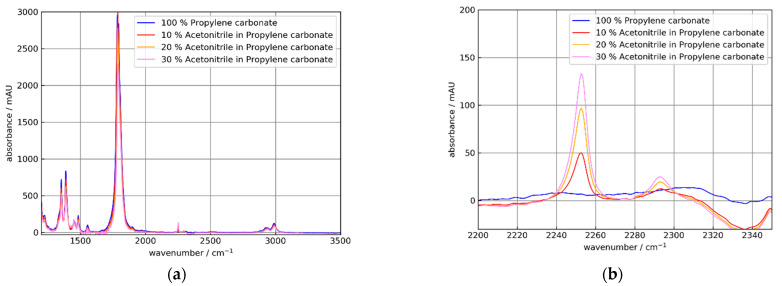
Absorbance spectra of 0, 10, 20, und 30% m/m solutions of acetonitrile (AC) in propylene carbonate (PC) using the uncoated Si ATR element C0. The absorbance spectra were referenced to air. (**a**) Broad spectral range showing strong PC absorption at 1782 cm^−1^ and relatively weak AC absorption at 2250 cm^−1^. (**b**) Detail showing AC absorption bands at 2250 and 2292 cm^−1^. The apparent negative absorption at 2340 cm^−1^ is an artifact caused by gaseous CO_2_ in the optical path.

**Figure 13:**
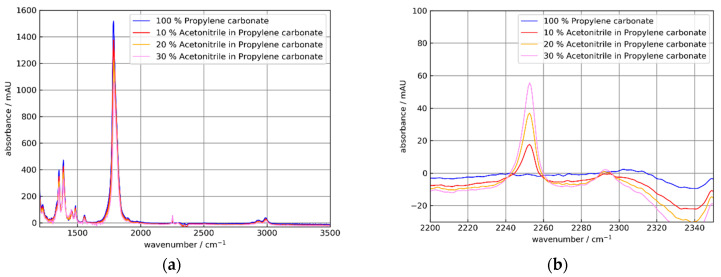
Absorbance spectra of 0, 10, 20, und 30% m/m solutions of acetonitrile (AC) in propylene carbonate (PC) using the Si ATR element C2. (**a**) Broad spectral range showing strong PC absorptions at 1388 and 1782 cm^−1^ and a weak AC absorption at 2250 cm^−1^. (**b**) Detail showing acetonitrile absorption bands at 2250 and 2292 cm^−1^. The absorbance spectra were referenced to air. The apparent negative absorption at 2340 cm^−1^ is caused by gaseous CO_2_.

The results of the measurements of AC in PC solutions for uncoated and NCD-coated Si ATR elements are shown in Figure 12 and Figure 13.

As expected from Figure 10, the absorbance for the coated crystal C2 was smaller than for the uncoated element C0. Additionally, the AC absorbance increased approximately linearly with the AC concentration. Respectively, the PC peak decreased similar to the PC concentrations. For further discussion, the peak absorbance data are collected in Table 3 and Table 4.

## 4. Discussion

It was shown that NCD-coated Si elements can serve as suitable materials for ATR process sensors. A thickness larger than approximately 300 nm was sufficient to ensure long operation time and stability of the surface sensor against cleaning agents. Compared to commonly used sapphire elements the long wavelength extreme of the spectral range was extended to approximately 8 µm.

Additionally, the results from Si hemispheres showed that with an angle of incidence of less than 34° and a suitable coating thickness, an amplification of the absorbances compared to uncoated Si elements could be achieved.

At larger angles of incidence, coating resulted in absorbance attenuation. For the strong water peak at 3300 cm^−1^, the data in Table 3 yield absorbance ratios of 0.61 for H2/H0 and 0.37 for H1/H0 as a result of the thicker coating of H1. The corresponding ratio for C1/C0 in Table 4 from Figure 11 is 0.38, indicating that the NCD thickness value reported by the manufacturer was too low. Experimental data were best fit by simulations assuming a layer thickness of 310 nm. Additionally, both absorbance ratios for water and PC in Table 3 fit well to the calculations in Figure 6a,b.

Comparing the water absorbances of the ATR crystals and the hemispheres yielded C0/H0 = 8.78, clearly showing the absorbance increase by multiple reflections. For the coated elements, a similar value of C1/H1 = 9 was obtained. This effective number of reflections was lower than the geometrically determined reflection number of 11 for the 45° elements. This discrepancy was attributed to intrinsic absorption, beam divergence, and scattering effects in the ATR element. (The strong PC absorbance at 1782 cm^−1^ saturation for the C0 signal reduced the value to ≈7).

A closer look at the data in Table 4 showed that the absorbance ratio of coated to uncoated Si elements C2/C0 moderately decreased from ≈0.6 at 1388 cm^−1^ to ≈0.4 at 3300 cm^−1^ within the experimental uncertainties (e.g., saturation effects for the strong PC absorption band at 1782 cm^−1^ and baseline corrections). This observation agreed qualitatively with the calculation in Figure 6b.

The absorbance increase at 2252 cm^−1^ with the AC concentration was slightly nonlinear. This was expected from previous work [13]. A similar behavior was found for the corresponding decrease of the PC peaks with increasing AC concentrations.

To indicate the potential of such diamond-coated Si ATR elements the following line of argument may be used: since ZnSe and diamond have almost the same MIR indices of refraction, for a bulk diamond crystal with identical dimensions the same absorbance is expected. Using a ZnSe element, ATR spectra of mixtures of AC in isopropanol were measured in [13] using the same FTIR setup with 11 reflections. There, for a 20% m/m mixture an absorbance of 155 mAU was determined, i.e., 14 mAU per reflection. Thus, for a diamond probe with two reflections one would expect 28 mAU.

With the NCD-coated Si element C2 a value of 44 mAU for a 20% m/m mixture of AC in PC (see Table 3) was obtained. Thus, with nine effective reflections a higher sensitivity than a diamond tip probe with two reflections were achieved.

Making use of the amplification effect by selection of an incidence angle of, e.g., 30° and a coating thickness of 0.5 µm would yield higher single reflection absorbance and an increase in the number of reflections. This would clearly outperform the sensitivity of a diamond tip probe.

## 5. Conclusions and Outlook

As a result, NCD-coated Si elements may be used in typical ATR accessories for FTIR instruments when a higher mechanical and/or chemical resistance of the surface is required. This is especially the case when using a compact FTIR instrument in a process environment.

Furthermore, the advent of compact MIR FPI micro spectrometers [29] opens the way for palm-sized process spectrometers using coated planar Si ATR elements as a cost-effective alternative to bulk diamond ATR crystals. By optimizing the angle of incidence and using suitable AR coatings for the entrance and exit faces of the Si ATR element, a substantially higher sensitivity can be expected owing to the high number of reflections.

Photometric detection is another possibility to realize a compact and robust process sensor as described, e.g., in Lambrecht et al. [13] and Theuer et al. [30]. A further step toward miniaturization is the use of MEMS technologies, as described in De Graaf et al. [31]. Radiation sources and thermopile detector elements can be fabricated using Si MEMS technology on a first wafer. A second Si wafer with the coated ATR element can then be bonded to the first wafer. The entire unit can be hermetically sealed in a compact housing, similar to infrared detector packages. A major advantage of using Si as an ATR element is that the processing and packaging steps are well established and ready for scale-up to high production volumes.

First experiments using such MEMS-integrated devices showed the feasibility of this approach. This topic will be further investigated. Although in several process applications the thermal and mechanical constraints will not allow inline use of such devices, there are still many applications left for cost-effective ATR sensors that can be fabricated in substantial volumes. Possible applications currently performed with FTIR transmission spectroscopy include, e.g., quality control of lubricating oil [32] or milk analysis [33]. Compact ATR sensors can be used for fast at-line analysis and can also be incorporated into handheld instruments.

Instead of using nanocrystalline diamond films as coating material, DLC coating could be an alternative [34]. Our first experiments with DLC-coated Si ATR elements showed that similar results can be obtained and that sufficient mechanical and chemical resistance of the DLC coating can be achieved. A major advantage of DLC is that coating on Ge ATR elements can be realized. Until now, diamond coating on Ge with the HFCVD process was only possible with a metallic bonding layer, which is detrimental for optical applications. Optimization of DLC-coated Ge ATR elements can be performed in a similar way to Si. With DLC-coated Ge an even broader spectral range and access to the MIR fingerprint range for robust, cost-effective, and compact ATR process sensors is foreseeable.

## Figures and Tables

**Table 1:** Silicon hemispheres (radius 12.5 mm) and silicon ATR crystals (50 mm × 10 mm × 2 mm; the short faces are wedged at a 45° angle). The thickness data of H1, H2 were optically determined by IPM. Data for C1, C2 were reported by the manufacturer based on the coating process parameters. Due to the spatial inhomogeneity of the coatings (see Figure 7b), a 10% uncertainty is assumed for all coating values.

Sample	Type	Coating Thickness/nm
H0	hemisphere	uncoated
H1	hemisphere	390
H2	hemisphere	<200
C0	ATR-crystal	uncoated
C1	ATR-crystal	200
C2	ATR-crystal	300

**Table 2:** Absorbance ratios for incidence angles of 30°, 35°, and 43° for water and propylene carbonate (PC) measurements from Figure 10 and for simulation results from Figure 6b.

Angle of Incidence/°	Absorbance Ratio H1/H0 for Water @ 3300 cm^−1^		Absorbance Ratio H1/H0 for PC @ 1782 cm^−1^	
	Measurement (390 nm)	Simulation (400 nm)	Measurement (390 nm)	Simulation (400 nm)
30°	1.40	1.23	0.90	1.03
35°	1.03	0.85	0.81	0.85
43°	0.48	0.39	0.61	0.60

**Table 3:** Peak absorbance values for uncoated and coated Si hemispheres at 45° incidence angle (data from Figure 10).

	Water Absorbance Peak @ 3300 cm^−1^/mAU	PC Absorbance Peak @ 1782 cm^−1^/mAU
H0 (uncoated)	140	430
H2 (<200 nm)	86	340
H1 (390 nm)	52	240
Absorbance ratio H2/H0	0.61	0.79
Absorbance ratio H1/H0	0.37	0.56

**Table 4:** Peak absorbance values for uncoated and coated Si ATR crystals for different NCD thickness values (ATR elements from Table 1, data from Figure 11, Figure 12 and Figure 13). Values in brackets are probably affected by saturation.

	Water Absorbance @ 3300 cm^−1^/mAU	AC Absorbance @ 2252 cm^−1^/mAU				PC Absorbance @ 1782 cm^−1^/mAU				PC Absorbance Peak @ 1388 cm^−1^/mAU			
AC in PC conc. (% m/m)	-	0	10	20	30	0	10	20	30	0	10	20	30
C0 (uncoated)	1230	-	50	96	133	(≈3000)	(≈3000)	(2765)	(2280)	800	689	602	542
C1 (≈200 nm)	470	-											
C2 (300 nm)	-	-	23	44	64	1513	1375	1222	1060	464	422	380	328
Absorbance ratio C1/C0 resp.C2/C0	0.38	-	0.46	0.46	0.48	(0.5)	(0.46)	(0.44)	(0.46)	0.58	0.61	0.63	0.60
